# Rhinovirus infection results in stronger and more persistent genomic dysregulation: Evidence for altered innate immune response in asthmatics at baseline, early in infection, and during convalescence

**DOI:** 10.1371/journal.pone.0178096

**Published:** 2017-05-26

**Authors:** Peter W. Heymann, Huyen-Tran Nguyen, John W. Steinke, Ronald B. Turner, Judith A. Woodfolk, Thomas A. E. Platts-Mills, Lisa Martin, Hua He, Jocelyn Biagini Myers, Mark Lindsey, Umasundari Sivaprasad, Mario Medvedovic, Naim Mahi, Holliday Carper, Deborah D. Murphy, James Patrie, Gurjit K. Khurana Hershey

**Affiliations:** 1 Division of Allergy, Immunology and Respiratory Medicine, Department of Pediatrics, University of Virginia, Charlottesville, Virginia, United States of America; 2 Department of Internal Medicine, Asthma and Allergic Diseases Center, University of Virginia, Charlottesville, Virginia, United States of America; 3 Division of Asthma Research, Cincinnati Children’s Hospital Medical Center, Department of Pediatrics, University of Cincinnati, Cincinnati, Ohio, United States of America; 4 Division of Allergy and Immunology, Cincinnati Children’s Hospital Medical Center, Department of Pediatrics, University of Cincinnati, Cincinnati, Ohio, United States of America; 5 Division of Infectious Diseases, Department of Pediatrics, University of Virginia, Charlottesville, Virginia, United States of America; 6 Division of Human Genetics, Cincinnati Children’s Hospital Medical Center, Department of Pediatrics, University of Cincinnati, Cincinnati, Ohio, United States of America; 7 Department of Environmental Health, University of Cincinnati, Cincinnati, Ohio, United States of America; 8 Department of Public Health Sciences, University of Virginia, Charlottesville, Virginia, United States of America; Imperial College London, UNITED KINGDOM

## Abstract

**Background:**

Rhinovirus (HRV) is associated with the large majority of virus-induced asthma exacerbations in children and young adults, but the mechanisms remain poorly defined.

**Methods:**

Asthmatics and non-asthmatic controls were inoculated with HRV-A16, and nasal epithelial samples were obtained 7 days before, 36 hours after, and 7 days after viral inoculation. RNA was extracted and subjected to RNA-seq analysis.

**Results:**

At baseline, 57 genes were differentially expressed between asthmatics and controls, and the asthmatics had decreased expression of viral replication inhibitors and increased expression of genes involved in inflammation. At 36 hours (before the emergence of peak symptoms), 1329 genes were significantly altered from baseline in the asthmatics compared to 62 genes in the controls. At this time point, asthmatics lacked an increase in IL-10 signaling observed in the controls. At 7 days following HRV inoculation, 222 genes were significantly dysregulated in the asthmatics, whereas only 4 genes were dysregulated among controls. At this time point, the controls but not asthmatics demonstrated upregulation of SPINK5.

**Conclusions:**

As judged by the magnitude and persistence of dysregulated genes, asthmatics have a substantially different host response to HRV-A16 infection compared with non-asthmatic controls. Gene expression differences illuminate biologically plausible mechanisms that contribute to a better understanding of the pathogenesis of HRV-induced asthma exacerbations.

## Introduction

Human rhinovirus (HRV) infection has been associated with the majority of asthma exacerbations in pediatric patients and with frequent loss of symptom control among asthmatic adults [1–3]. HRV is a positive-sense, single-stranded picornavirus that is subcategorized into A, B, and C strains, with HRV-A and HRV-C genotypes implicated in most exacerbations [4]. The mechanism for the propensity of HRV infection to trigger an asthma exacerbation remains ill-defined.

After 3 years of age, most asthma exacerbations caused by HRV occur in those who are atopic. Moreover, the risk for wheezing with HRV is strongly associated with high levels of total and allergen specific IgE and with the presence of Th2 related airway inflammation prior to an infection [5–7]. Some studies suggest that decreased interferon production in response to HRV infection in the setting of Th2 inflammation may contribute to asthma exacerbation [8]. For example, HRV infection of cultured asthmatic bronchial epithelial cells induced less type I interferon production and resistance to early apoptosis compared to control cells, and this was associated with increased viral replication [9]. Further, decreased production of type I and III interferons in bronchoalveolar lavage cells has been associated with more severe exacerbations in adult asthmatics [10]. Yet, a genome-wide expression analysis of HRV-infected primary bronchial epithelial cells did not reveal any significant differences in interferon expression related to asthma [11].

Following viral exposure, we postulate that gene expression at the epithelial cell level is the earliest response to HRV that, in turn, initiates and influences subsequent events that influence the clinical outcome. Indeed, the presence of Th2 associated inflammation (e.g., increased levels of FeNO and eosinophil cationic protein [ECP]) detected in the asthmatic airway) has been proposed to contribute to HRV-induced asthma exacerbation during seasons of increased allergen exposure [7]. Epithelial cells of the asthmatic airway also have an increased number of protease-activating receptors (PAR). The activation of such receptors leads to opening of tight junctions, production of cytokines and chemokines, and degranulation of eosinophils and mast cells [12]. Taken together, we hypothesize that the host response to HRV in the asthmatic airway will be different at the time of initial virus exposure and lead to a unique signature of gene expression that will improve our understanding of asthma attacks caused by HRV.

## Experimental procedures

### Patient characteristics

The participants included 5 adults with mild asthma (mean age 25 years; range = 20 to 33 years) and 5 non-atopic adults without asthma (mean age 21.4 years; range = 20 to 23 years). They were screened and characterized with respect to lung function, atopy, and their asthmatic status prior to enrollment (results shown in Table 1). Inclusion and exclusion criteria were similar to our previous experimental challenges with HRV-A16 [7]. In brief, all asthmatic subjects had physician-diagnosed, mild asthma and used only inhaled bronchodilators for symptom control. Those using inhaled steroids, nasal steroids, cromolyn, nedocromil sodium, ipratropium bromide, or leukotriene modifiers within one month prior to enrollment were excluded, because these medications could alter epithelial cell gene expression and clinical outcome. In keeping with the diagnosis of mild asthma, those who had used oral steroids within 6 weeks prior to enrollment or who were hospitalized or needed treatment in the emergency room for asthma within 3 years of enrollment were excluded. Asthma subjects were also excluded if they had received allergen immunotherapy within the last 3 years or if their ACT score, to judge symptom control during the month before virus inoculation, was less than 19. All asthmatic subjects were atopic as judged by positive skin prick tests (i.e., a response 3 mm greater than a saline control) to common aeroallergens using extracts from Greer Pharmaceuticals (Lenoir, NC). The allergens included dust mite (*D*. *farinae* and *D*. *pteronyssinus*), cockroach, cat, dog, *Alternaria*, *Aspergillus*, *Penicillium*, 7 grass mix, Bermuda grass, Eastern tree mix, and ragweed. Each asthmatic subject had a positive methacholine challenge at a concentration ≤ 16mg/mL.

**Table 1:** Patient characteristics.

Group	Age	Sex	FEV1% Predicted	Total IgE	Skin Tests	ACT Score
**Asthmatics**	20	F	82	726	Dust mite, cat, dog, mold, tree, grass	24
	25	M	75	623	Dust mite, cat, dog, mold, tree, grass	21
	33	F	97	1045	Dust mite, tree, grass, ragweed	21
	26	F	104	1429	Dust mite, cat, dog, tree, grass	22
	21	M	94	1989	Dust mite, cat, tree, grass, ragweed	23
**Controls**	20	M	72	18.3		25
	22	F	104	5.2		25
	20	F	96	34.9		25
	23	F	101	23		25
	22	M	104	42.5		25

Non-asthmatic control subjects were individuals with no history of asthma or allergic disease. They were excluded if they had a positive methacholine challenge test or a positive skin prick test to any of the aeroallergens tested. All subjects (asthmatics and controls) were excluded if 1) they had a positive test for serum neutralizing antibody to HRV-A16, 2) they had chronic heart or lung disease (other than asthma), or 3) other chronic illnesses such as primary or secondary immunodeficiency disorders. They were also excluded if they had a 5 pack/year history of smoking or any smoking within the last 6 months, or symptoms of upper or lower respiratory infection during the 6 weeks prior to virus inoculation. The study was approved by the Institutional Review Board at the University of Virginia, and all participants signed informed consent before enrollment.

### Evaluation at enrollment and virus inoculation

Questionnaires focused on each participant’s past and present medical history were administered at enrollment along with a physical exam, spirometry, methacholine challenge, and a nasal wash to confirm absence of HRV infection through qPCR and culture. A blood sample was obtained to re-evaluate serum neutralizing antibody to HRV-A16 and a total IgE level. After a 7-day run-in period to monitor baseline values, each participant was inoculated on arrival at a hotel where subjects were isolated in their own room and monitored daily over a 4-day period after inoculation. The inoculation pool of HRV-A16 was prepared according to GMP specifications and approved by the FDA for experimental challenges (IND # 15162). Each participant was inoculated with a total dose of 300 TCID^50^mL (tissue culture infectious dose 50/ml) after doing a nasal wash at baseline to test for the presence of pre-existing infection with HRV by culture and qPCR.

### Study design

**Fig 1:**
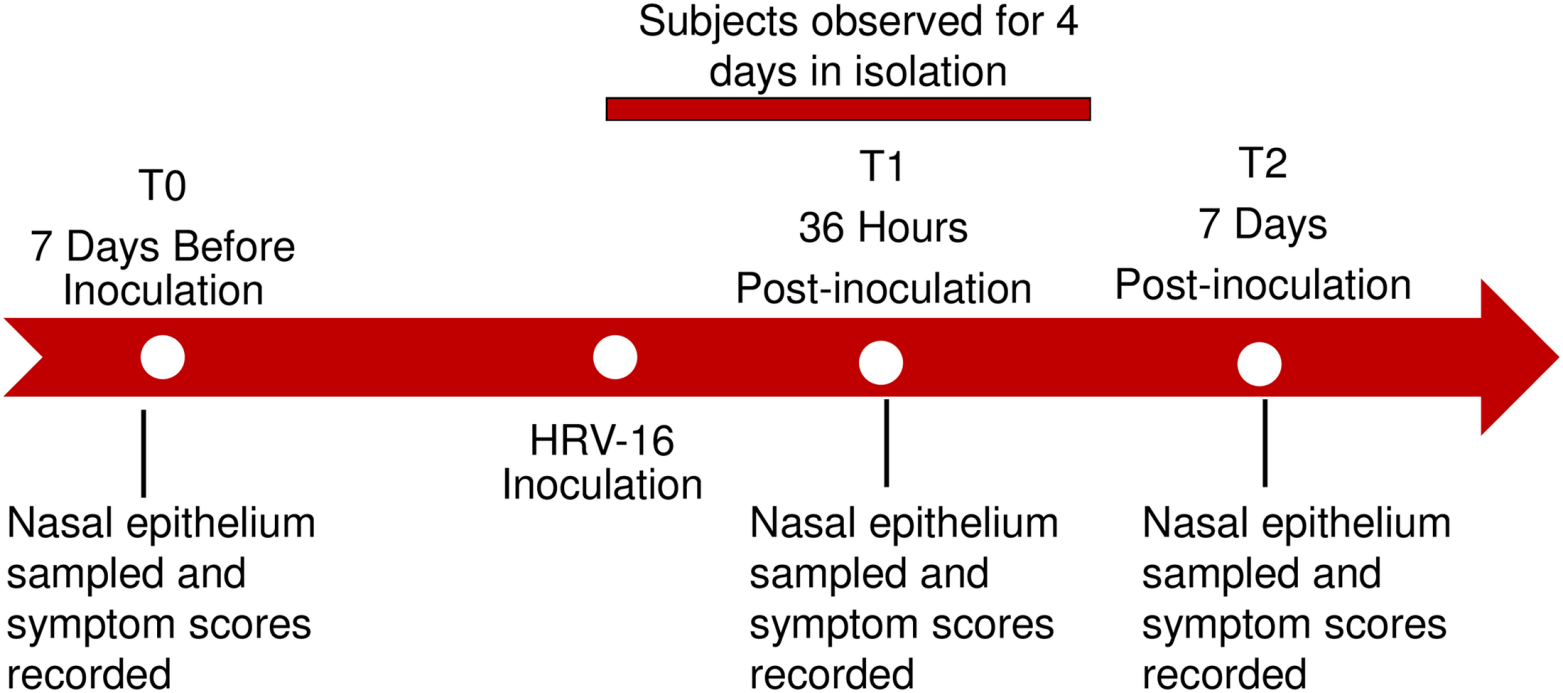
Timeline of study. RNA-seq analysis was applied to all nasal epithelial samples.

The study design and timeline is shown in Fig 1. Nasal epithelial samples were obtained after nasal washes (to remove mucous) by gentle scraping of the inferior turbinates within each nostril visualized with a head lamp and a Bionix^®^, Toledo, OH. disposable nasal speculum. The scrapings were obtained with an ASI RhinoPro^®^, Arlington Scientific, Inc., Springville, UT. This procedure was done at enrollment (7 days before inoculation (T0)) and at 36 hours after inoculation (T1) in the hotel. A final scraping was obtained 7 days after virus inoculation (T2) during a clinic visit planned at follow-up. RNA extractions from the epithelial cell scrapings were done within 2 hours using the Qiagen AllPrep DNA/RNA Mini Kit^®^, Hilden, Germany. The nasal washes done prior to the nasal scrapings were used to evaluate viral load and were performed as previously described [5, 13].

Upper airway symptoms were assessed daily on diary cards using the modified Jackson criteria to monitor symptoms of rhinorrhea, sneezing, nasal congestion, sore throat, headache, chills/fevers, and watery/itchy eyes using a scale of 1 through 3, with 1 being mild and clearly present and 3 being severe and interfering with activities [14]. Lower airway symptoms of cough, shortness of breath, chest discomfort, wheezing, and night awakenings were also reported by participants on diary cards daily throughout the study using the same severity scale.

### Bioinformatic RNA-seq data analysis

RNA was isolated from the nasal epithelial samples at the University of Virginia and sent to the University of Cincinnati College of Medicine Genomics, Epigenomics, and Sequencing Core for analysis via RNA-seq. Sequence reads were aligned to the reference human genome (hg19) using the TopHat aligner [15]. Reads aligning to each known transcript were counted and all follow up analyses were performed using Bioconductor packages for next-generation sequencing data analysis [16]. The differential gene expression analysis was performed based on the negative-binomial statistical model of read counts as implemented in the *edgeR* Bioconductor package [17]. Genes differentially expressed between asthma and control samples before inoculation (time point T0) were identified using the simple generalized linear model with the single factor (asthma = yes or no). Genes differentially expressed after inoculation (time points T1 and T2) in comparison to the baseline (time point T0) were identified by fitting a generalized linear model with both time and subject factors to account for the subject-to-subject variability. These comparisons were made separately for asthma and control samples. P-values were adjusted for multiple testing using the false discovery rates and differential expressions with FDR-adjusted p-value of less than 0.05 were considered statistically significant [18]. To elucidate the most common pathways affected the by gene dysregulation, all genes dysregulated at least 1.5 fold were analyzed with QIAGEN’s Ingenuity^®^ Pathway Analysis (IPA^®^, QIAGEN Redwood City, www.qiagen.com/ingenuity).

### Upper and lower respiratory tract symptom score analyses

Cumulative upper and lower respiratory tract symptom (CURTS and CLRTS, respectively) scores were derived from diary cards. The CURTS and CLRTS scores were computed by numerically adding up the individual upper and lower respiratory tract symptom scores recorded daily by study participants from day 0 (day of HRV-A16 inoculation) to post-inoculation day 7 (T2). The CURTS and CLRTS scores were analyzed by way of negative binomial generalized linear models. Study-group (i.e. the asthmatic or non-asthmatic group) served as the *independent* variable of each analysis and between-group comparisons were focused on comparing the means of the underlying cumulative symptom score distributions. A two-sided p≤0.05 decision rule was used as the *between* study-group comparison null hypothesis rejection criterion. The software of the PROC GENMOD procedure of SAS version 9.4 (SAS Institute Inc., Cary, NC) was used to conduct the cumulative symptom score analysis.

## Results

### Tests for atopy, Rhinovirus infection, and symptoms

**Fig 2:**
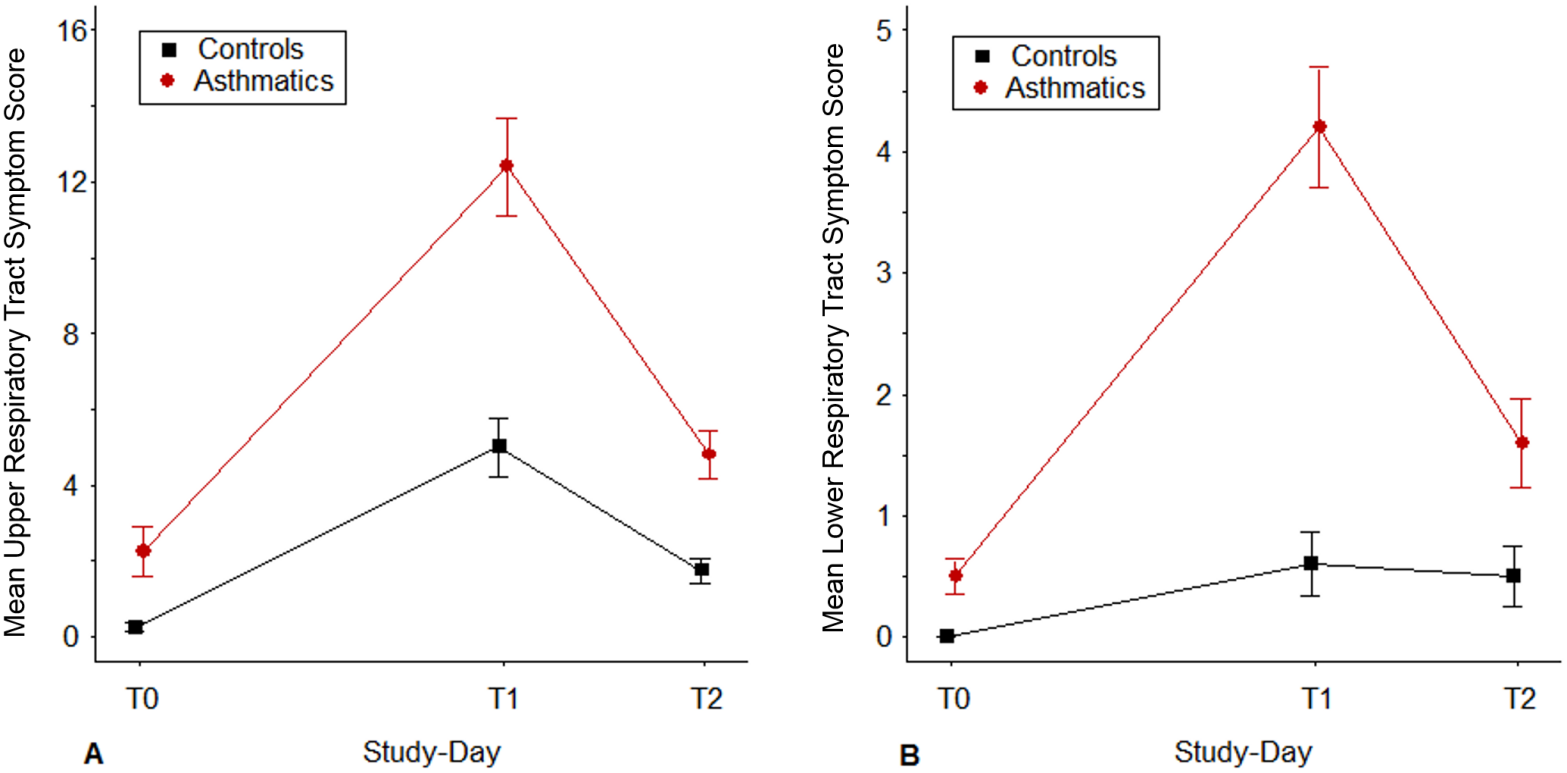
Mean upper respiratory tract symptom score (A) and mean lower respiratory tract symptom score (B). Points identify the mean symptom scores determined (as described in the Methods) on each day (T0, T1, T2) when epithelial cell scrapings were obtained, Vertical lines identify the range of values within ± 1 standard error of the mean total symptom score.

The five asthmatic participants in this study had 4 or more positive tests for allergen specific IgE by prick skin testing and high levels of total IgE (geometric mean [GM] = 1061 IU/ml; range = 623–1989) (Table 1). The 5 controls had negative skin tests and their total IgE levels were low (GM = 20 IU/ml; range = 5–42). Following virus inoculation, HRV titers in cultures from nasal washes were quantified with average viral titers from T0 to T2 ranging from 0.92 to 1.96 titer/mL from the asthmatics. Three of the five controls had HRV isolated in culture, with average viral titers from T0 to T2 ranging from 1.74 to 2.03 titer/mL. The remaining 2 control subjects did not have positive viral cultures, but they had low levels of HRV-A16 detected through qPCR during the study to confirm successful inoculation. The mean CURTS scores over the 7 days of monitoring after RV inoculation were 65 (34–126, 95% confidence limits [CL]) and 32 (16–62, 95% CL) for the asthmatic and non-asthmatic groups, respectively; p = 0.133 (Fig 2). The mean CLRTS scores over the same time period were 25 (10–64; 95% CL) and 3 (1.0–9; 95% CL) for the asthmatics and non-asthmatic controls, respectively; p = 0.004.

### Differences in gene expression between asthmatics and controls evident at baseline

**Fig 3:**
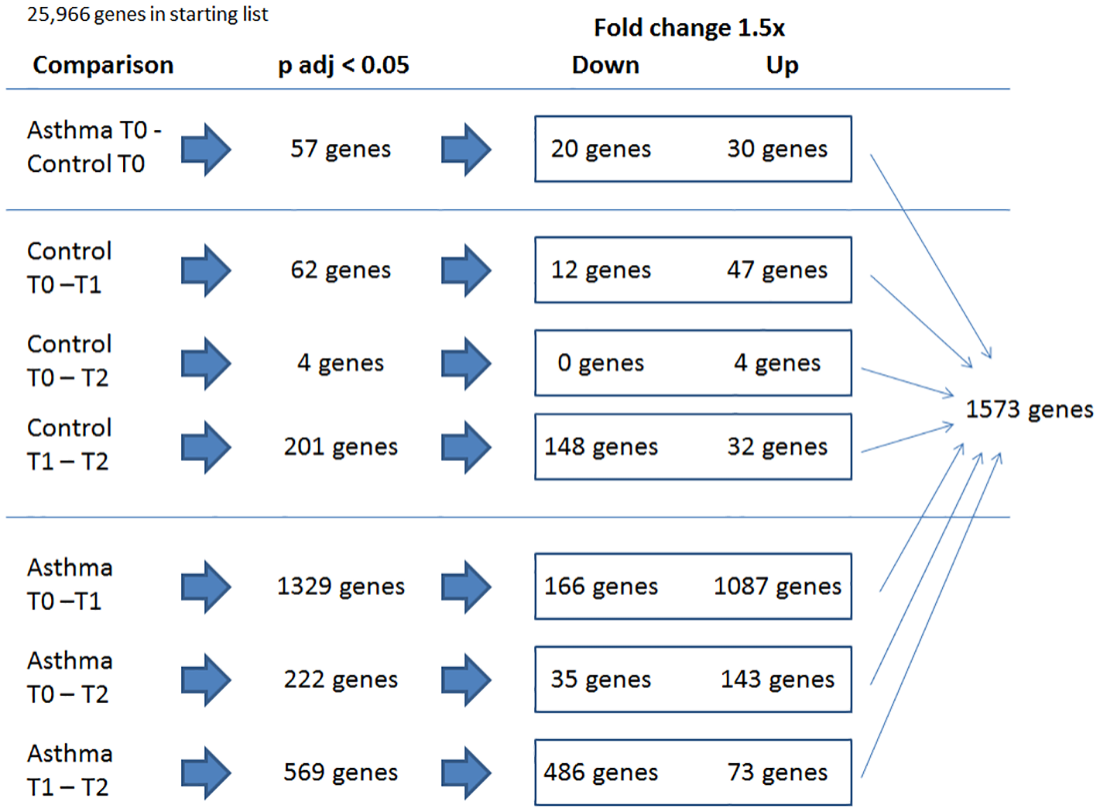
Dysregulated genes in asthmatic and control subjects.

Fig 3 is an overview of the gene dysregulation in asthmatics and controls at baseline and in response to HRV infection. At baseline, 50 genes were differentially expressed at least 1.5 fold in asthmatics compared to controls (Table 2). Asthmatics had increased expression of genes involved in inflammation, including interleukin-1 receptor, type 1 (*IL1R1*), arachidonate 5-lipoxygenase (*ALOX5*), and tryptase alpha/beta-1 (*TPSAB1*). Additionally, asthmatics had decreased expression of viral replication inhibitors—interferon-induced protein with tetratricopeptide repeats 1 (*IFIT1*) and SAM domain and HD domain-containing protein 1 (*SAMHD1*).

**Table 2:** Top 30 genes with expression affected at least 3 fold between asthmatics and controls at baseline.

geneid	symbol	name	padj	fold change
**2624**	GATA2	GATA binding protein 2	0.0212	66.3627
**1359**	CPA3	carboxypeptidase A3 (mast cell)	0.0400	64.8943
**64499**	TPSB2	tryptase beta 2 (gene/pseudogene)	0.0003	52.4161
**7177**	TPSAB1	tryptase alpha/beta 1	0.0003	51.6452
**6422**	SFRP1	secreted frizzled-related protein 1	0.0363	25.5794
**10631**	POSTN	periostin, osteoblast specific factor	0.0472	21.2008
**2354**	FOSB	FBJ murine osteosarcoma viral oncogene homolog B	0.0006	17.3689
**1958**	EGR1	early growth response 1	0.0212	10.2780
**2353**	FOS	FBJ murine osteosarcoma viral oncogene homolog	0.0212	10.1764
**4915**	NTRK2	neurotrophic tyrosine kinase, receptor, type 2	0.0145	7.3831
**3164**	NR4A1	nuclear receptor subfamily 4, group A, member 1	0.0396	6.3766
**240**	ALOX5	arachidonate 5-lipoxygenase	0.0212	6.3736
**9021**	SOCS3	suppressor of cytokine signaling 3	0.0386	6.0078
**6550**	SLC9A3	solute carrier family 9, subfamily A (NHE3, cation proton antiporter 3), member 3	0.0158	5.9875
**7832**	BTG2	BTG family, member 2	0.0039	5.4052
**55107**	ANO1	anoctamin 1, calcium activated chloride channel	0.0000	5.1866
**857**	CAV1	caveolin 1, caveolae protein, 22kDa	0.0212	4.5104
**7538**	ZFP36	ZFP36 ring finger protein	0.0181	4.0869
**9245**	GCNT3	glucosaminyl (N-acetyl) transferase 3, mucin type	0.0000	4.0763
**1549**	CYP2A7	cytochrome P450, family 2, subfamily A, polypeptide 7	0.0341	4.0437
**56165**	TDRD1	tudor domain containing 1	0.0080	-16.3343
**1.01E+08**	LOC100996579	uncharacterized LOC100996579	0.0341	-5.6716
**8647**	ABCB11	ATP-binding cassette, sub-family B (MDR/TAP), member 11	0.0039	-5.0905
**285313**	IGSF10	immunoglobulin superfamily, member 10	0.0212	-4.4396
**347**	APOD	apolipoprotein D	0.0474	-4.2888
**345930**	ECT2L	epithelial cell transforming 2 like	0.0212	-4.1564
**2628**	GATM	glycine amidinotransferase (L-arginine:glycine amidinotransferase)	0.0158	-4.0163
**23007**	PLCH1	phospholipase C, eta 1	0.0133	-3.8777
**3434**	IFIT1	interferon-induced protein with tetratricopeptide repeats 1	0.0084	-3.8357
**730101**	LOC730101	uncharacterized LOC730101	0.0212	-3.7865

### Rhinovirus-16 infection results in increased number of dysregulated genes and more persistent gene dysregulation in asthmatics compared to controls

**Fig 4:**
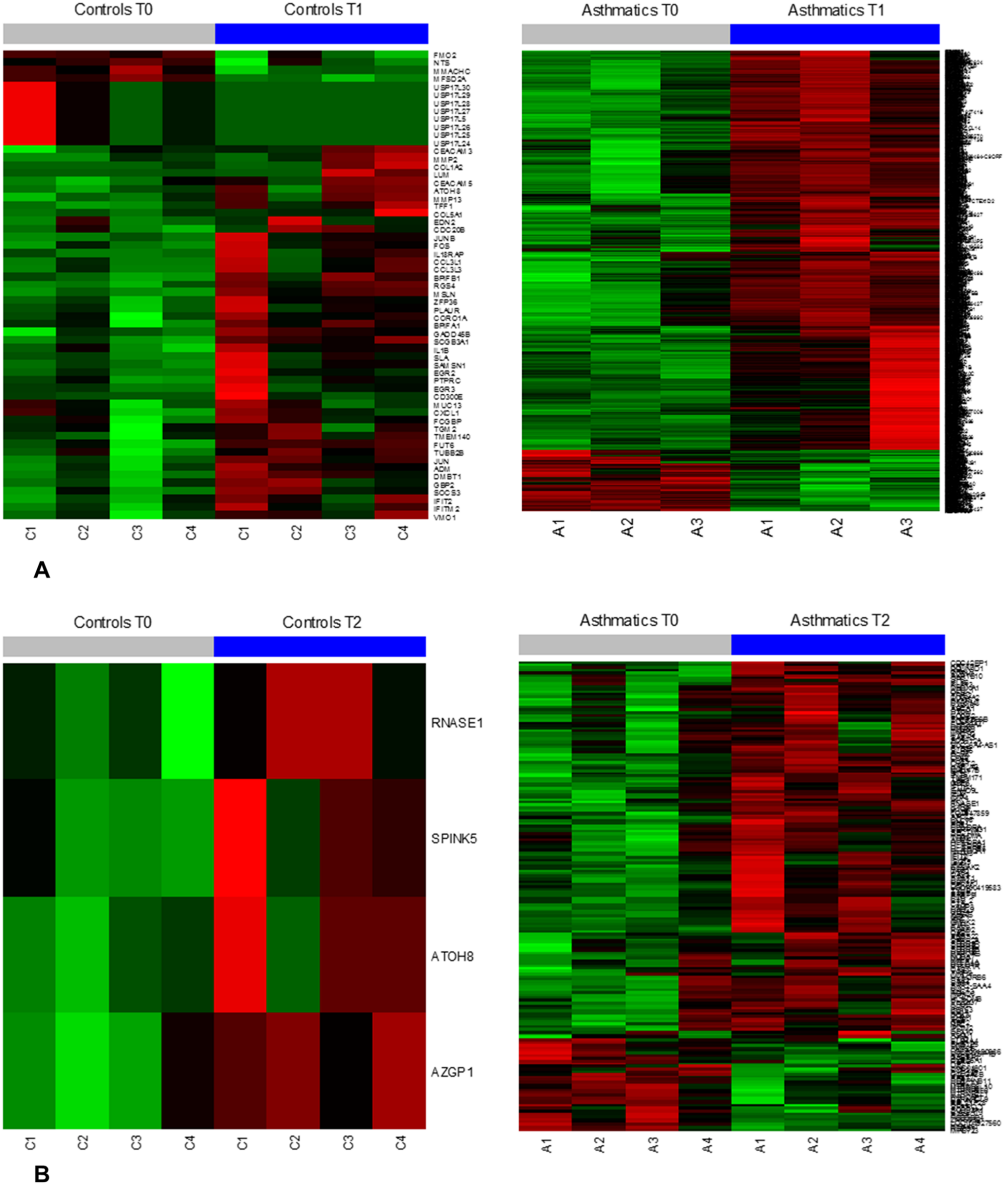
A. Heat map illustrating gene dysregulation in controls (left) and asthmatics (right) at T1 versus T0. The baseline samples of one asthmatic and one control patient were not of sufficient quality and were not included in analysis. The sample from T1 of one asthmatic was not of sufficient quality and was also excluded. B. Heat maps illustrating gene dysregulation in controls (left) and asthmatics (right) at T2 versus T0. The normalized RNA-seq raw counts for SPINK5 in controls at baseline and at T2 ranged from 2.6 to 35.3 and are reflected in the heat map with green representing lower values and red representing higher values.

**Fig 5:**
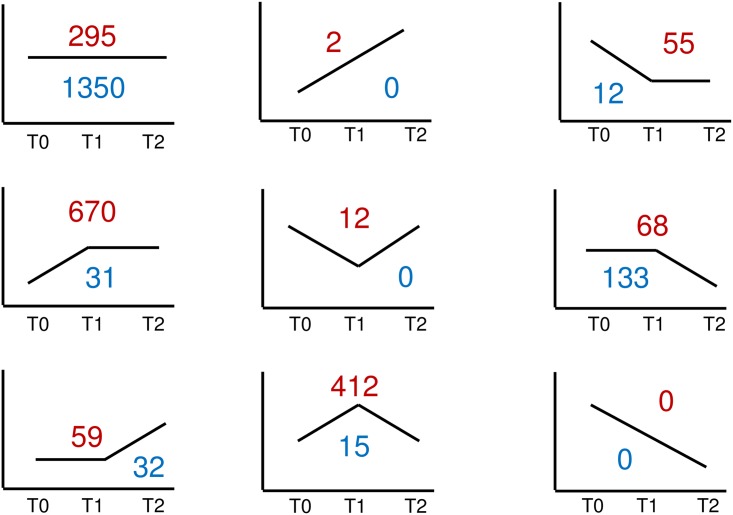
Cluster analysis of gene expression asthmatics (red) and controls (blue). A total of 1573 genes had at least 1.5 fold change in controls or asthmatics with significant dysregulation (p adj < 0.05). 1350 of these genes were unchanged in controls. 295 of these genes were unchanged in asthmatics. 670 genes were upregulated from T0 to T1 and unchanged from T1 to T2 in asthmatics. 412 genes were upregulated from T0 to T1 and downregulated from T1 to T2 in asthmatics.

As evident in Fig 3, there is substantial gene dysregulation in response to HRV infection in both asthmatics and controls, but it is stronger and more persistent in asthmatics. Following inoculation with HRV, gene expression was significantly altered at T1 in asthmatics and controls with changes favoring upregulation in both groups. However, the asthmatic group showed significantly more dysregulated genes compared to controls. From T0 to T1, there were 1253 genes dysregulated at least 1.5 fold in asthma participants compared to 59 genes in controls (Fig 4A). By T2, gene expression in controls had essentially returned to baseline levels with only 4 genes dysregulated at least 1.5 fold compared to baseline expression (Fig 4B). In contrast, asthmatics continued to have significant gene dysregulation with 178 genes upregulated or downregulated at least 1.5 fold from baseline even 7 days later (Fig 4B). The gene expression from T0 through T2 in asthmatics and controls were categorized into 9 different clusters (Fig 5). A total of 1573 genes were found to have at least 1.5 fold change in expression in all participants of the study—asthmatics or controls—and to be significantly dysregulated (p adj < 0.05). Of this total, 1350 genes were unchanged in controls. Controls had 133 genes that were unchanged from T0 to T1 that then were downregulated from T1 to T2; 31 genes were upregulated from T0 to T1 and then were unchanged from T1 to T2. Asthmatics, on the other hand, had only 295 genes that were unchanged from T0 through T2. 670 genes were upregulated from T0 to T1 and were unchanged from T1 to T2 in asthmatics; 412 genes were upregulated from T0 to T1 and then downregulated from T1 to T2. This cluster analysis further illustrates the increased magnitude and persistence of gene dysregulation in asthmatics compared to controls in response to HRV infection.

### The biology of the response to Rhinovirus-16 infection is different in asthmatics compared to controls

We next more carefully examined the differences in the biologic response to HRV-A16 in asthmatics versus controls. Tables 3–6 list the 30 most dysregulated genes in asthmatics and controls at T1 versus T0 and T2 versus T0 (complete listing of dysregulated genes is detailed in S1 File). Pathway analyses revealed that most of the dysregulation in both asthmatics and controls involves the innate and early adaptive immune response to HRV infection including IL-6 signaling [*FOS*, *IL18RAP*, *IL1B*, and *JUN*], dendritic cell maturation [including *ICAM1*, *IL1RN*, *JAK2*, *STAT1*,*STAT4*, *TNF*, *TLR2*, *TLR4*, *NFKB2*, and *PIK3R5*], granulocyte adhesion/diapedesis [including *ICAM1*, *ITGB2*, *CCL3L1*, *CCL3L3*, *CXCL1*, *MMP2*, *MMP13*, *C5AR1*, *CLDN10*, and *CXCR4*], interferon signaling [*STAT1*, *JAK2*, *IFIT1*, *IFIT3*, *IFITM1*, *IFITM2*, *IFITM3*, *OAS1*, and *TAP1*], and B-cell development [including *CD19*, *IL7R*, *CD86*, *PTPRC*, *HLA-DMA*, *HLA-DMB*, *HLA-DOA*, *HLA-DQA*, *HLA-DRA*, and *HLA-DRB1*]. There were several notable differences between asthmatics and controls. The regulatory IL-10 pathway was upregulated in controls at T1, with significant increase in expression of *SOCS3*, a suppressor of cytokine signaling. This increase in IL-10 signaling was not evident in asthmatics at T1 or T2. Also, in controls, *SPINK5*, which has been implicated in epithelial maintenance and repair, was induced 5.3 fold at T2 compared to baseline expression. This increase in SPINK5 was not evident in asthmatics.

**Table 3:** Genes with expression affected at least 3 fold in control group from T0 to T1.

geneid	symbol	name	padj	fold change
**4060**	LUM	lumican	0.020943	72.8022
**342510**	CD300E	CD300e molecule	0.008487	38.3880
**3553**	IL1B	interleukin 1, beta	0.003612	21.7846
**1278**	COL1A2	collagen, type I, alpha 2	0.000348	21.7134
**414062**	CCL3L3	chemokine (C-C motif) ligand 3-like 3	0.026042	17.5723
**6349**	CCL3L1	chemokine (C-C motif) ligand 3-like 1	0.026042	17.5723
**1960**	EGR3	early growth response 3	0.0124	12.4203
**5329**	PLAUR	plasminogen activator, urokinase receptor	0.033343	9.6363
**1959**	EGR2	early growth response 2	0.048586	9.2946
**4313**	MMP2	matrix metallopeptidase 2 (gelatinase A, 72kDa gelatinase, 72kDa type IV collagenase)	0.00553	7.4013
**1289**	COL5A1	collagen, type V, alpha 1	0.043756	7.2466
**9021**	SOCS3	suppressor of cytokine signaling 3	1.27E-06	7.1697
**64092**	SAMSN1	SAM domain, SH3 domain and nuclear localization signals 1	0.026322	7.1446
**2353**	FOS	FBJ murine osteosarcoma viral oncogene homolog	0.022499	6.7704
**6503**	SLA	Src-like-adaptor	0.017101	6.2597
**5999**	RGS4	regulator of G-protein signaling 4	0.012113	5.8468
**5788**	PTPRC	protein tyrosine phosphatase, receptor type, C	0.030499	5.2359
**84913**	ATOH8	atonal homolog 8 (Drosophila)	0.007565	4.2610
**8807**	IL18RAP	interleukin 18 receptor accessory protein	0.012113	4.2399
**133**	ADM	adrenomedullin	0.009749	3.8967
**51297**	BPIFA1	BPI fold containing family A, member 1	0.005879	3.8852
**11151**	CORO1A	coronin, actin binding protein, 1A	0.012414	3.8204
**1907**	EDN2	endothelin 2	0.015707	3.8022
**92304**	SCGB3A1	secretoglobin, family 3A, member 1	2.08E-08	3.6013
**7538**	ZFP36	ZFP36 ring finger protein	0.012292	3.3842
**4322**	MMP13	matrix metallopeptidase 13 (collagenase 3)	0.12414	3.2228
**7031**	TFF1	trefoil factor 1	0.001698	3.0703

**Table 4:** Top 30 genes with expression affected at least 3 fold in asthmatics from T0 to T1.

geneid	symbol	name	padj	fold change
**3552**	IL1A	interleukin 1, alpha	1.71E-05	28.92492495
**402665**	IGLON5	IgLON family member 5	0.002964315	26.55078527
**719**	C3AR1	complement component 3a receptor 1	4.17E-05	26.09216702
**4973**	OLR1	oxidized low density lipoprotein (lectin-like) receptor 1	0.000737414	25.21195402
**6373**	CXCL11	chemokine (C-X-C motif) ligand 11	2.34E-10	22.43688977
**4064**	CD180	CD180 molecule	0.038699948	21.74115708
**23601**	CLEC5A	C-type lectin domain family 5, member A	0.000460589	20.90985398
**6347**	CCL2	chemokine (C-C motif) ligand 2	0.001554308	20.54486259
**2865**	FFAR3	free fatty acid receptor 3	1.65E-05	18.54119076
**101928513**	CATIP-AS1	CATIP antisense RNA 1	0.005560741	15.54512675
**5806**	PTX3	pentraxin 3, long	1.99E-07	14.73660174
**6351**	CCL4	chemokine (C-C motif) ligand 4	3.02E-10	13.67747639
**160622**	GRASP	GRP1 (general receptor for phosphoinositides 1)-associated scaffold protein	0.008466801	13.67246691
**9560**	CCL4L2	chemokine (C-C motif) ligand 4-like 2	7.20E-07	13.43126991
**388372**	CCL4L1	chemokine (C-C motif) ligand 4-like 1	9.04E-07	13.33820322
**5329**	PLAUR	plasminogen activator, urokinase receptor	7.87E-08	13.28421895
**10578**	GNLY	granulysin	0.04395143	12.75855934
**221692**	PHACTR1	phosphatase and actin regulator 1	0.016054301	12.71174252
**3576**	CXCL8	chemokine (C-X-C motif) ligand 8	1.35E-09	12.70191061
**3575**	IL7R	interleukin 7 receptor	2.51E-05	12.57533077
**10563**	CXCL13	chemokine (C-X-C motif) ligand 13	0.041383665	12.1909315
**56165**	TDRD1	tudor domain containing 1	0.000775926	12.09246229
**100128385**	FAM225B	family with sequence similarity 225, member B (non-protein coding)	0.030859801	11.91060151
**8013**	NR4A3	nuclear receptor subfamily 4, group A, member 3	0.001564312	11.82961672
**7772**	ZNF229	zinc finger protein 229	0.046632528	11.7866035
**1493**	CTLA4	cytotoxic T-lymphocyte-associated protein 4	0.033675632	11.71145859
**400680**	LINC00664	long intergenic non-protein coding RNA 664	2.79E-06	11.59893421
**23769**	FLRT1	fibronectin leucine rich transmembrane protein 1	0.003228573	-23.25413453
**25787**	DGCR9	DiGeorge syndrome critical region gene 9 (non-protein coding)	0.020863424	-18.17084882
**152**	ADRA2C	adrenoceptor alpha 2C	0.002794832	-13.37477796

**Table 5:** Genes with expression affected at least 3 fold in control group from T0 to T2.

geneid	symbol	name	padj	fold change
**84913**	ATOH8	atonal homolog 8 (Drosophila)	0.034015	3.796982
**6035**	RNASE1	ribonuclease, RNase A family, 1 (pancreatic)	0.018146	5.157555
**11005**	SPINK5	serine peptidase inhibitor, Kazal type 5	1.51E-08	5.268909

**Table 6:** Top 30 Genes with expression affected at least 3 fold in asthmatics from T0 to T2.

geneid	symbol	name	padj	fold change
**10578**	GNLY	granulysin	0.023905745	19.41766265
**5079**	PAX5	paired box 5	0.024519067	18.71066511
**2537**	IFI6	interferon, alpha-inducible protein 6	5.10E-06	15.51483751
**4283**	CXCL9	chemokine (C-X-C motif) ligand 9	0.043209055	8.507631831
**5551**	PRF1	perforin 1 (pore forming protein)	0.024519067	7.640101617
**91543**	RSAD2	radical S-adenosyl methionine domain containing 2	9.98E-12	7.566978504
**3434**	IFIT1	interferon-induced protein with tetratricopeptide repeats 1	0.024776552	7.008492872
**10964**	IFI44L	interferon-induced protein 44-like	0.001195079	5.75513534
**6362**	CCL18	chemokine (C-C motif) ligand 18 (pulmonary and activation-regulated)	0.0162948	5.623785255
**9636**	ISG15	ISG15 ubiquitin-like modifier	7.68E-05	5.215923291
**5653**	KLK6	kallikrein-related peptidase 6	0.019548496	4.868135902
**129607**	CMPK2	cytidine monophosphate (UMP-CMP) kinase 2, mitochondrial	0.002773796	4.741074774
**3437**	IFIT3	interferon-induced protein with tetratricopeptide repeats 3	0.002006977	4.276974551
**710**	SERPING1	serpin peptidase inhibitor, clade G (C1 inhibitor), member 1	0.000537538	4.177324231
**79931**	TNIP3	TNFAIP3 interacting protein 3	0.000432613	4.105550274
**80320**	SP6	Sp6 transcription factor	0.02127551	4.077235267
**27074**	LAMP3	lysosomal-associated membrane protein 3	0.008830902	3.862185131
**348**	APOE	apolipoprotein E	4.41E-06	3.846620987
**7453**	WARS	tryptophanyl-tRNA synthetase	0.000534318	3.822353718
**100126311**	MIR147B	microRNA 147b	4.68E-18	3.652618463
**8771**	TNFRSF6B	tumor necrosis factor receptor superfamily, member 6b, decoy	0.000551508	3.643040835
**3433**	IFIT2	interferon-induced protein with tetratricopeptide repeats 2	0.003161937	3.640824157
**4481**	MSR1	macrophage scavenger receptor 1	0.013181546	3.578676195
**641517**	DEFB109P1B	defensin, beta 109, pseudogene 1B	0.030905088	-27.09924
**284654**	RSPO1	R-spondin 1	0.006456265	-5.278907
**101927560**	LOC101927560	uncharacterized LOC101927560	4.11E-05	-4.316873
**100616209**	MIR4461	microRNA 4461	7.68E-05	-4.285838
**128102**	HSD3BP4	hydroxy-delta-5-steroid dehydrogenase, 3 beta, pseudogene 4	0.002899914	-4.12954
**1592**	CYP26A1	cytochrome P450, family 26, subfamily A, polypeptide 1	0.000130986	-3.80011
**102465432**	MIR6723	microRNA 6723	0.027519068	-3.601905

## Discussion

**Fig 6:**
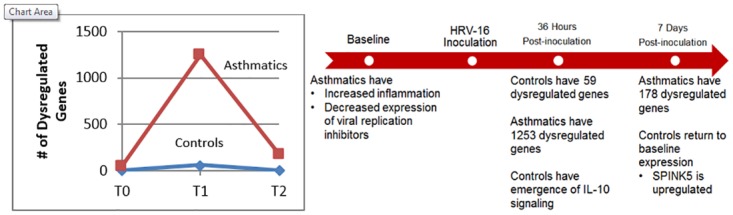
Summary of gene dysregulation in asthmatics versus controls from T0 to T2 (left) and summary of differences in innate immunity, including the epithelial barrier (right). These differences may contribute to increased magnitude and persistence of gene dysregulation in asthmatics in response to HRV infection.

Our data demonstrate that the biology of the host response to HRV is fundamentally different in asthmatics compared to controls with striking differences in epithelial cell gene expression during the early, innate phase of the infection before symptoms peak. Asthmatics demonstrated increased magnitude and persistence of gene dysregulation, as well as distinct differences in the quality of the response following infection with HRV-A16 (Fig 6).

Consistent with reports that increased levels of inflammatory biomarkers are detected in the upper and lower airway of asthmatics before an experimental challenge with HRV [7], baseline differences in gene expression associated with inflammatory pathways were also significantly increased among the asthmatics compared to the controls. These baseline differences may contribute to an increased susceptibility to viral infection in keeping with the observation that asthmatics had decreased expression of viral replication inhibitors compared to controls. We observed in asthmatics increased expression of genes involved in inflammation—interleukin-1 receptor, type 1 (*IL1R1*), arachidonate 5-lipoxygenase (*ALOX5*), and tryptase alpha/beta-1 (*TPSAB1*). Tryptase is an activator of PAR-2, which in turn causes weakening of tight junctions within the epithelial layer, allowing for easier access to receptors used by HRV to infect epithelial cells and contributing to the severity and persistence of symptoms [12, 19].

As with prior studies, gene expression in both asthmatics and controls skewed towards activation of the innate and early adaptive immune response, including interferon signaling and granulocyte adhesion/diapedesis, after HRV inoculation [20]. Additionally, pathway analysis showed an early emergence of IL-10 signaling pathway in the control group from T0 to T1, which was absent in the asthmatic group from T0 to T1 and even from T0 to T2. While nasal mucosal samples do not exclude infiltrating cells such as monocytes, a major producer of IL-10, Guajardo et al has previously shown that such samples are composed mainly (> 92%) of respiratory epithelial cells [21]. IL-10 is a regulatory cytokine with dominant immunosuppressive effects resulting in inhibition of IL-12 production, reduced expression of co-stimulatory molecules and Class I MHC, suppression of production of pro-inflammatory cytokines including IL-2, TNF-alpha, and IL-5, and increased production of anti-inflammatory cytokines including IL-1Ralpha and VEGF [22]. Indeed, impaired IL-10 responses have been associated with HRV-induced asthma exacerbations [23]. Further, several studies have shown that polymorphisms in the IL-10 promoter are associated with asthma, possibly due to decreased IL-10 production [24–28].

Although the asthmatics had markedly persistent gene dysregulation following HRV infection, there were a few genes that were notably induced during the convalescent recovery phase (T2) in control subjects. One of 4 genes that was induced in controls at T2 compared to baseline was *SPINK5*, which has been intensely studied in asthma due to its location within chromosome 5q31-33, a region shown in genome-wide linkage scans to be linked to asthma and atopy [29]. *SPINK5* encodes an inhibitor of multiple serine proteases, including plasmin, neutrophil elastase, and trypsin, and mutations in this gene have been associated with Netherton syndrome [29]. *SPINK5* polymorphisms/haplotypes have also been associated with asthma susceptibility in numerous populations [30–32]. The upregulation of *SPINK5* in controls at T2 suggests it may be important in epithelial repair. While a recent study involving the transfection of A549 cells (adenocarcinomic human alveolar basal epithelial cells) with SPINK5 expression vectors resulted in increased production of IL-6, IL-8, and RANTES, transfection also resulted in increased susceptibility to cell death [33]. As destruction of infected cells is key for antigen presentation in the immune response to active viral infections, increased *SPINK5* expression may be a protective measure. Additionally, studies measuring SPINK5 in nasal epithelial tissue revealed that low SPINK5 expression was associated with chronic rhinosinusitis with and without nasal polyps [34] and among those with chronic rhinosinusitis and aspirin intolerance [35]. The association between atopic diseases and polymorphisms or decreased expression of *SPINK5* suggests a significant role for this protease inhibitor in epithelial cell maintenance and repair.

The RV challenge model has been helpful in other investigations to explore mechanistic questions focused on the asthmatic response to RV [11, 23, 36–38]. To our knowledge, however, this is the first report of changes in gene expression in response to HRV observed well before symptoms peak among asthmatics who, in our study, experienced more significant lower respiratory tract symptoms and sustained gene expression that was still augmented compared to the non-asthmatics 7 days after virus inoculation. Our study was limited by a small sample size, and the gene expression analysis could not be completed for two participants—one control and one asthmatic—due to samples of insufficient quality. Because of the broad inclusion and exclusion criteria required to find subjects who are eligible for participation, it is difficult to enroll larger numbers of mild asthmatics who are seronegative to the strain of virus used for inoculation and who are not using controller medications in these studies. However, the dysregulation of genes noted at 36 hours, and the persistent changes at 7 days, was striking among the asthmatics and these results are unlikely to be altered by including data from more subjects. For example, epithelial cell gene expression has been examined in response to HRV-A16 in nasal scrapings from healthy, non-asthmatic subjects who tested positive for HRV in cultures of their nasal washes. As compared to the 1263 genes dysregulated at least 1.5 fold among the asthmatics in our study, significantly fewer genes were dysregulated in nasal scrapings at 48 hours in that study (i.e., 471 genes showed a ≥ 2-fold increase in gene expression and 201 genes decreased ≥ 0.5-fold) [39]

In the future, further studies would be desirable to examine gene expression in epithelial cell samples obtained at earlier time points after HRV inoculation. A longer follow-up period (e.g., 3 to 4 weeks after inoculation) would also be beneficial to determine how long the gene dysregulation in asthmatics persists and to evaluate any differences in relation to the adaptive immune response. Additionally, the enrollment of atopic subjects without asthma would be of interest, along with selected samples collected for gene expression analysis from the lower airway.

In summary, asthmatics have a substantially altered host response to HRV-A16 infection in terms of magnitude, quality, and persistence when compared to non-asthmatic controls. Differences in gene expression were apparent at baseline, during infection, and during the onset of convalescence/repair. At baseline, increased expression of inflammatory genes and decreased expression of viral replication inhibitors was evident in asthmatics, and emergence of IL-10 signaling early in infection was evident only in controls. During convalescence/repair, upregulation of *SPINK5* was seen only in controls underscoring the role of this protease inhibitor in the recovery phase. These key differences provide novel mechanistic insights into how HRV infection may contribute to asthma exacerbation and highlight previously unrecognized pathways that may be important targets for therapeutic intervention.

## Supporting information

S1 File Complete list of dysregulated genes. This excel workbook details the complete list of dysregulated genes in both asthmatics and controls.
(XLSX)

## References

[1] Bizzintino J, Lee WM, Laing IA, Vang F, Pappas T, Zhang G, et al Association between human rhinovirus C and severity of acute asthma in children. Eur Respir J. 2011;37(5):1037–42. 10.1183/09031936.00092410 20693244 PMC3024467

[2] Khetsuriani N, Kazerouni NN, Erdman DD, Lu X, Redd SC, Anderson LJ, et al Prevalence of viral respiratory tract infections in children with asthma. The Journal of allergy and clinical immunology. 2007;119(2):314–21. 10.1016/j.jaci.2006.08.041 17140648 PMC7112359

[3] Gern JE. How rhinovirus infections cause exacerbations of asthma. Clin Exp Allergy. 2015;45(1):32–42. 10.1111/cea.12428 25270551

[4] Lee WM, Lemanske RF Jr, Evans MD, Vang F, Pappas T, Gangnon R, et al Human rhinovirus species and season of infection determine illness severity. American journal of respiratory and critical care medicine. 2012;186(9):886–91. 10.1164/rccm.201202-0330OC 22923659 PMC3530215

[5] Heymann PW, Kennedy JL. Rhinovirus-induced asthma exacerbations during childhood: the importance of understanding the atopic status of the host. The Journal of allergy and clinical immunology. 2012;130(6):1315–6. 10.1016/j.jaci.2012.10.024 23195527 PMC4379703

[6] Soto-Quiros M, Avila L, Platts-Mills TAE, Hunt JF, Erdman DD, Carper H, et al High Titers of IgE Antibody to Dust Mite Allergen and the Risk for Wheezing Among Asthmatic Children Infected with Rhinovirus. The Journal of allergy and clinical immunology. 2012;129(6):1499–505.e5. 10.1016/j.jaci.2012.03.040 22560151 PMC3792652

[7] Zambrano JC, Carper HT, Rakes GP, Patrie J, Murphy DD, Platts-Mills TA, et al Experimental rhinovirus challenges in adults with mild asthma: response to infection in relation to IgE. The Journal of allergy and clinical immunology. 2003;111(5):1008–16. 12743565 10.1067/mai.2003.1396

[8] Durrani SR, Montville DJ, Pratt AS, Sahu S, DeVries MK, Rajamanickam V, et al Innate immune responses to rhinovirus are reduced by the high-affinity IgE receptor in allergic asthmatic children. Journal of Allergy and Clinical Immunology. 2012;130(2):489–95. 10.1016/j.jaci.2012.05.023 22766097 PMC3437329

[9] Wark PA, Johnston SL, Bucchieri F, Powell R, Puddicombe S, Laza-Stanca V, et al Asthmatic bronchial epithelial cells have a deficient innate immune response to infection with rhinovirus. The Journal of experimental medicine. 2005;201(6):937–47. 10.1084/jem.20041901 15781584 PMC2213100

[10] Contoli M, Message SD, Laza-Stanca V, Edwards MR, Wark PA, Bartlett NW, et al Role of deficient type III interferon-lambda production in asthma exacerbations. Nature medicine. 2006;12(9):1023–6. 10.1038/nm1462 16906156

[11] Bochkov YA, Hanson KM, Keles S, Brockman-Schneider RA, Jarjour NN, Gern JE. Rhinovirus-induced modulation of gene expression in bronchial epithelial cells from subjects with asthma. Mucosal immunology. 2010;3(1):69–80. 10.1038/mi.2009.109 19710636 PMC2884103

[12] Reed CE, Kita H. The role of protease activation of inflammation in allergic respiratory diseases. The Journal of allergy and clinical immunology. 2004;114(5):997–1008; quiz 9. 10.1016/j.jaci.2004.07.060 15536399

[13] Kennedy JL, Shaker M, McMeen V, Gern J, Carper H, Murphy D, et al Comparison of viral load in individuals with and without asthma during infections with rhinovirus. American journal of respiratory and critical care medicine. 2014;189(5):532–9. 10.1164/rccm.201310-1767OC 24471509 PMC3977713

[14] Jackson GG, Dowling HF, Spiesman IG, Boand AV. Transmission of the common cold to volunteers under controlled conditions. I. The common cold as a clinical entity. AMA archives of internal medicine. 1958;101(2):267–78. 13497324 10.1001/archinte.1958.00260140099015

[15] Trapnell C, Pachter L, Salzberg SL. TopHat: discovering splice junctions with RNA-Seq. Bioinformatics (Oxford, England). 2009;25(9):1105–11. 10.1093/bioinformatics/btp120 PMC2672628 19289445

[16] Huber W, Carey VJ, Gentleman R, Anders S, Carlson M, Carvalho BS, et al Orchestrating high-throughput genomic analysis with Bioconductor. Nature methods. 2015;12(2):115–21. 10.1038/nmeth.3252 25633503 PMC4509590

[17] Anders S, McCarthy DJ, Chen Y, Okoniewski M, Smyth GK, Huber W, et al Count-based differential expression analysis of RNA sequencing data using R and Bioconductor. Nature protocols. 2013;8(9):1765–86. 10.1038/nprot.2013.099 23975260

[18] Storey JD, Tibshirani R. Statistical significance for genomewide studies. Proceedings of the National Academy of Sciences of the United States of America. 2003;100(16):9440–5. 10.1073/pnas.1530509100 12883005 PMC170937

[19] Ubl JJ, Grishina ZV, Sukhomlin TK, Welte T, Sedehizade F, Reiser G. Human bronchial epithelial cells express PAR-2 with different sensitivity to thermolysin. American journal of physiology Lung cellular and molecular physiology. 2002;282(6):L1339–48. 10.1152/ajplung.00392.2001 12003791

[20] Bosco A, Ehteshami S, Panyala S, Martinez FD. Interferon regulatory factor 7 is a major hub connecting interferon-mediated responses in virus-induced asthma exacerbations in vivo. The Journal of allergy and clinical immunology. 2012;129(1):88–94. 10.1016/j.jaci.2011.10.038 22112518 PMC3246116

[21] Guajardo JR, Schleifer KW, Daines MO, Ruddy RM, Aronow BJ, Wills-Karp M, et al Altered gene expression profiles in nasal respiratory epithelium reflect stable versus acute childhood asthma. The Journal of allergy and clinical immunology. 2005;115(2):243–51. 10.1016/j.jaci.2004.10.032 15696077

[22] Stanic B, van de Veen W, Wirz OF, Ruckert B, Morita H, Sollner S, et al IL-10-overexpressing B cells regulate innate and adaptive immune responses. The Journal of allergy and clinical immunology. 2015;135(3):771–80.e8. 10.1016/j.jaci.2014.07.041 25240783

[23] Message SD, Laza-Stanca V, Mallia P, Parker HL, Zhu J, Kebadze T, et al Rhinovirus-induced lower respiratory illness is increased in asthma and related to virus load and Th1/2 cytokine and IL-10 production. Proceedings of the National Academy of Sciences of the United States of America. 2008;105(36):13562–7. 10.1073/pnas.0804181105 18768794 PMC2528869

[24] Hobbs K, Negri J, Klinnert M, R LJ, Borish L. Interleukin-10 and transforming growth factor-beta promoter polymorphisms in allergies and asthma. American journal of respiratory and critical care medicine. 1998;158:1958–62. 10.1164/ajrccm.158.6.9804011 9847292

[25] Chatterjee R, Batra J, Kumar A, Mabalirajan U, Nahid S, Niphadkar PV, et al Interleukin-10 promoter polymorphisms and atopic asthma in North Indians. Clinical and experimental allergy: journal of the British Society for Allergy and Clinical Immunology. 2005;35(7):914–9. 16008678 10.1111/j.1365-2222.2005.02273.x

[26] Nie W, Fang Z, Li B, Xiu QY. Interleukin-10 promoter polymorphisms and asthma risk: a meta-analysis. Cytokine. 2012;60(3):849–55. 10.1016/j.cyto.2012.08.023 23017230

[27] Hyun MH, Lee CH, Kang MH, Park BK, Lee YH. Interleukin-10 promoter gene polymorphisms and susceptibility to asthma: a meta-analysis. PloS one. 2013;8(1):e53758 10.1371/journal.pone.0053758 23335974 PMC3546046

[28] Zheng XY, Guan WJ, Mao C, Chen HF, Ding H, Zheng JP, et al Interleukin-10 promoter 1082/-819/-592 polymorphisms are associated with asthma susceptibility in Asians and atopic asthma: a meta-analysis. Lung. 2014;192(1):65–73. 10.1007/s00408-013-9519-8 24162871

[29] Jongepier H, Koppelman GH, Nolte IM, Bruinenberg M, Bleecker ER, Meyers DA, et al Polymorphisms in SPINK5 are not associated with asthma in a Dutch population. The Journal of allergy and clinical immunology. 2005;115(3):486–92. 10.1016/j.jaci.2004.12.013 15753894

[30] Biagini Myers JM, Martin LJ, Kovacic MB, Mersha TB, He H, Pilipenko V, et al Epistasis between serine protease inhibitor Kazal-type 5 (SPINK5) and thymic stromal lymphopoietin (TSLP) genes contributes to childhood asthma. The Journal of allergy and clinical immunology. 2014;134(4):891–9.e3. 10.1016/j.jaci.2014.03.037 24831437 PMC4186896

[31] Kabesch M, Carr D, Weiland SK, von Mutius E. Association between polymorphisms in serine protease inhibitor, kazal type 5 and asthma phenotypes in a large German population sample. Clinical and Experimental Allergy. 2004;34(3):340–5. 15005725 10.1111/j.1365-2222.2004.01860.x

[32] Martinez-Aguilar NE, Del Rio-Navarro BE, Navarro-Olivos E, Garcia-Ortiz H, Orozco L, Jimenez-Morales S. SPINK5 and ADRB2 haplotypes are risk factors for asthma in Mexican pediatric patients. The Journal of asthma: official journal of the Association for the Care of Asthma. 2015;52(3):232–9. 25233048 10.3109/02770903.2014.966913

[33] Birben E, Sackesen C, Turgutoglu N, Kalayci O. The role of SPINK5 in asthma related physiological events in the airway epithelium. Respiratory medicine. 2012;106(3):349–55. 10.1016/j.rmed.2011.11.007 22133475

[34] Richer SL, Truong-Tran AQ, Conley DB, Carter R, Vermylen D, Grammer LC, et al Epithelial genes in chronic rhinosinusitis with and without nasal polyps. American journal of rhinology. 2008;22(3):228–34. 10.2500/ajr.2008.22.3162 18588753 PMC2810157

[35] Fruth K, Goebel G, Koutsimpelas D, Gosepath J, Schmidtmann I, Mann WJ, et al Low SPINK5 expression in chronic rhinosinusitis. The Laryngoscope. 2012;122(6):1198–204. 10.1002/lary.23300 22570283

[36] Adura PT, Reed E, Macintyre J, Del Rosario A, Roberts J, Pestridge R, et al Experimental rhinovirus 16 infection in moderate asthmatics on inhaled corticosteroids. The European respiratory journal. 2014;43(4):1186–9. 10.1183/09031936.00141713 24311773

[37] Beale J, Jayaraman A, Jackson DJ, Macintyre JD, Edwards MR, Walton RP, et al Rhinovirus-induced IL-25 in asthma exacerbation drives type 2 immunity and allergic pulmonary inflammation. Science translational medicine. 2014;6(256):256ra134 10.1126/scitranslmed.3009124 25273095 PMC4246061

[38] Jackson DJ, Makrinioti H, Rana BM, Shamji BW, Trujillo-Torralbo MB, Footitt J, et al IL-33-dependent type 2 inflammation during rhinovirus-induced asthma exacerbations in vivo. American journal of respiratory and critical care medicine. 2014;190(12):1373–82. 10.1164/rccm.201406-1039OC 25350863 PMC4299647

[39] Proud D, Turner RB, Winther B, Wiehler S, Tiesman JP, Reichling TD, et al Gene expression profiles during in vivo human rhinovirus infection: insights into the host response. American journal of respiratory and critical care medicine. 2008;178(9):962–8. 10.1164/rccm.200805-670OC 18658112

